# Supplementary material for: Rapid turnover of life-cycle-related genes in the brown algae
Source: Genome Biol. 2019 Feb 14;20:35. doi: 10.1186/s13059-019-1630-6 (PMC6374913; doi:10.1186/s13059-019-1630-6)
Supplement: Supplementary file 3 — Protein alignments, corresponding Gblocks html file and CDS sequences for the lineage conserved gametophyte-biased genes. (ZIP 2109 kb) [file 13059_2019_1630_MOESM3_ESM.zip › Con_GA_alignments/SJ02306.aa_ali.fasta-gb.htm]

SJ02306.aa.fasta.trans.aa\_ali.fasta


## Gblocks 0.91b Results

Processed file: **SJ02306.aa.fasta.trans.aa\_ali.fasta**  
Number of sequences: **2**  
Alignment assumed to be: **Protein**  
New number of positions: **209** (selected positions are underlined in blue)

```
                         10        20        30        40        50        60
                 =========+=========+=========+=========+=========+=========+
SJ02306          ELLRNNAMAALGQRFASAIEVLIQEMVRERDGKTSAPSSVSIASVRDSKGRRRPMADQNA
Mac.65779.1      ------------------------------------------------------------
                                                                             


                         70        80        90       100       110       120
                 =========+=========+=========+=========+=========+=========+
SJ02306          TTRSTGNAFGTGGLGSTGSEVSTATASKGSQESFLTGRGAGGGGGGSSAGVLPGGRGGCS
Mac.65779.1      ------------------------------------------------------------
                                                                             


                        130       140       150       160       170       180
                 =========+=========+=========+=========+=========+=========+
SJ02306          TTNETVGEAKERCKTSPLQGKMGRSRGNLGCRIEGRETTRKHHTVSAKRGHGLSSPPKVG
Mac.65779.1      ------------------------SRGNLGCRIEGRETTRKHHTVSAKRGHGISSPPKGG
                                         ####################################


                        190       200       210       220       230       240
                 =========+=========+=========+=========+=========+=========+
SJ02306          RASRDTVAMLNKFQFSKDALRRHQMSHESWIPEELRMKMMRRSMAVIKENVHESVAQRSL
Mac.65779.1      RTSRDTAGMLNKFQFSKDALRRHQMSHESWIPEELRMKMMRRSMAVIKENVHESVAQRSL
                 ############################################################


                        250       260       270       280       290       300
                 =========+=========+=========+=========+=========+=========+
SJ02306          VRKEKKRSSRSELEIVKTQEKFGTKHQRPCALCEQSFSEVNLVLAVPFKAIVDLRIAWQK
Mac.65779.1      VRKEKKRSSRSELEIVKTQEKFGTKHQRPCALCEQSFSEVNLVLAVPFKAIVDLRIAWQK
                 ############################################################


                        310       320       330       340       350       360
                 =========+=========+=========+=========+=========+=========+
SJ02306          TLPQAKGSTTKTNRTMLMPPQCYNEVK---------------------------------
Mac.65779.1      TLPQAKGSATKTNRTMLMPPQCYNEVKVCAFCSQLFHDPLGYRPTWDMEVSRMRKKREQL
                 ###########################                                 


                        370       380       390       400
                 =========+=========+=========+=========+======
SJ02306          --------------------ATQSKEQDQQVSPPDVAPSLSVEAAA
Mac.65779.1      ALQKEREYWDPLRNSSLNTLATQSKDQDQQVSPPDVAPPLSIETAA
                                     ##########################
```

```
Parameters used
Minimum Number Of Sequences For A Conserved Position: 2
Minimum Number Of Sequences For A Flanking Position: 2
Maximum Number Of Contiguous Nonconserved Positions: 8
Minimum Length Of A Block: 10
Allowed Gap Positions: None
Use Similarity Matrices: Yes
```

```
Flank positions of the 2 selected block(s)
Flanks: [145  327]  [381  406]  

New number of positions in SJ02306.aa.fasta.trans.aa_ali.fasta-gb:  209  (51% of the original 406 positions)
```
